# Hollow Fiber Supported Liquid Membrane Extraction Combined with HPLC-UV for Simultaneous Preconcentration and Determination of Urinary Hippuric Acid and Mandelic Acid

**DOI:** 10.3390/membranes7010008

**Published:** 2017-02-12

**Authors:** Abdulrahman Bahrami, Farhad Ghamari, Yadollah Yamini, Farshid Ghorbani Shahna, Abbas Moghimbeigi

**Affiliations:** 1 Excellence Centre of Occupational Health, Research Center for Health Sciences, School of Public Health, Hamadan University of Medical Sciences, Hamadan 6517838736, Iran; bahrami@umsha.ac.ir (A.B.); fghorbani@umsha.ac.ir (F.G.S.); 2 Department of Occupational Health, School of Public Health, Arak University of Medical Sciences, Arak 3819693345, Iran; 3 Department of Chemistry, Faculty of Sciences, Tarbiat Modares University, Tehran 1411713116, Iran; yyamini@modares.ac.ir; 4 Department of Biostatistics and Epidemiology, School of Public Health and Center of Health Research, Hamedan University of Medical Sciences, Hamedan 6517838736, Iran; moghimb@yahoo.com

**Keywords:** hippuric acid, mandelic acid, toluene and ethylbenzene metabolites, HF-LPME, facilitated pH gradient transport, HPLC-UV

## Abstract

This work describes a new extraction method with hollow-fiber liquid-phase microextraction based on facilitated pH gradient transport for analyzing hippuric acid and mandelic acid in aqueous samples. The factors affecting the metabolites extraction were optimized as follows: the volume of sample solution was 10 mL with pH 2 containing 0.5 mol·L^−1^ sodium chloride, liquid membrane containing 1-octanol with 20% (*w*/*v*) tributyl phosphate as the carrier, the time of extraction was 150 min, and stirring rate was 500 rpm. The organic phase immobilized in the pores of a hollow fiber was back-extracted into 24 µL of a solution containing sodium carbonate with pH 11, which was placed inside the lumen of the fiber. Under optimized conditions, the high enrichment factors of 172 and 195 folds, detection limit of 0.007 and 0.009 µg·mL^−1^ were obtained. The relative standard deviation (RSD) (%) values for intra- and inter-day precisions were calculated at 2.5%–8.2% and 4.1%–10.7%, respectively. The proposed method was successfully applied to the analysis of these metabolites in real urine samples. The results indicated that hollow-fiber liquid-phase microextraction (HF-LPME) based on facilitated pH gradient transport can be used as a sensitive and effective method for the determination of mandelic acid and hippuric acid in urine specimens.

## 1. Introduction

Environmental and occupational exposure to the aromatic solvents toluene and ethylbenzene is frequent because these compounds are commonly used as solvents in making paints, dyes, paint thinners, rubber, adhesives, pharmaceutical products and plastic materials. They are also added to gasoline along with other aromatic compounds to improve octane ratings. So, the general population is potentially exposed to these toxic solvents [1,2].

Several epidemiological and toxicological studies on the harmful and adverse effects of these toxicants, such as functional alterations of the central nervous system, immune, kidney, liver, and reproductive effects, have been conducted [2,3,4]. Increased risk of some cancers such as leukemia and gastrointestinal cancers has been associated with chronic exposure to toluene and ethylbenzene [5]. Due to increasing concern over exposure to these toxic chemicals in ambient air and occupational environments, biological and environmental monitoring is important to evaluate adverse effects and potential risk hazards. The toluene and ethylbenzene are metabolized via oxidization and conjugation with glycine to hippuric acid (HA) and mandelic acid (MA) respectively and excreted in urine [4]. The urinary concentration of these metabolites is directly related to the amount of external exposure and absorbed dose of these compounds. American Conference of Governmental Industrial Hygienists (ACGIH) has recommended these biomarkers as the biological monitoring of exposure to toluene and ethylbenzene [6]. Since the urinary concentration of these metabolites in environmental and low-level occupational are very low, development and implementation of a sensitive and selective method for determination of these compounds is essential. 

Several analytical methods, including GC-MS, HPLC-UV, HPLC/MS/MS, and CE have been used for the analysis of HA and MA in urine [2,7,8,9,10,11,12,13].

Some conventional sample preparation techniques such as liquid–liquid extraction (LLE) and solid-phase extraction (SPE) have been used to perform sample clean-up before the analysis of these metabolites in biological fluids [2,7,13,14,15,16]. However, applications of these techniques have some drawbacks. They are a multi-step and long procedures, which lead to analyte losses; they consume large volumes of high-purity solvents (in some cases they are expensive, toxic, or highly polluting), providing low pre-concentration [17,18,19,20,21]. Recently, a novel microextraction technique called hollow-fiber liquid-phase microextraction (HF-LPME) has been used in different environmental and biological samples for different analytes to overcome these problems [17,20,21,22,23,24,25,26,27]. In environmental application, both HF-LPME in its two- and three-phase modes have been used to determine various types of contaminants include pesticides, insecticides, aromatic amines, BTEX, phenolic compounds, polychlorinated biphenyls, polynuclear aromatic hydrocarbons, muconic acid and drugs in soil, water and wastewater matrices [17,23,28,29,30,31,32,33]. In order to increase the extraction of hydrophilic compounds from the aqueous donor phase into the organic phase, carrier mediated liquid phase microextraction has been used as an active transport mode [19]. However, there has to date been no similar study based on the application of the HF-LPME method for the determination of HA and MA in human urine. In the present study, the carrier-mediated three-phase hollow fiber microextraction method followed by high performance liquid chromatography-ultra violet was used to develop a method for extraction, pre-concentration and determination of MA and HA in human urine. In order to obtain better extraction performance and maximum sensitivity, important parameters that might affect the extraction efficiency were investigated and optimized.

Finally, a simple protocol of the HF-LPME based on facilitated pH gradient transport method was represented for the assessment of toluene and ethylbenzene exposure.

## 2. Experimental

### 2.1. Reagents and Materials

HA, MA, methanol-gradient grade, ethyl acetate, 1-decanol, trioctylamine (TOA), tributyl phosphate (TBP), trioctylphosphine oxide (TOPO), n-dodecane, 1-octanol, sodium chloride, sodium carbonate, acetic acid (glacial), and hydrochloric acid were obtained from Merck (Darmstadt, Germany). Dihexyl ether was purchased from Sigma-Aldrich (Darmstadt, Germany).

Synthetic urine as the blank urine was provided by Fisher Scientific (Pittsburgh, PA, USA). Water was purified with a Millipore Milli-Q system (Bedford, MA, USA) before use. The Q3/2 Accurel PP polypropylene hollow-fiber (HF) membranes (200 µm wall thickness, 600 µm i.d., 0.2 µm pore size) were obtained from Membrana GmbH (Wuppertal, Germany).

### 2.2. Preparation of Standard Solutions and Real Samples

The stock standard solution of each metabolite (1 g·L^−1^) was prepared by dissolving 10 mg of the standard in 10 mL of methanol–water (1:1) and stored in a fridge at 4 °C until use. The working solutions containing two interest analytes were prepared daily by diluting the appropriate amount of the stock solution with water prior to use. Human urine samples were collected from a healthy person (no exposure to organic solvents) and exposed workers (petrol station workers and car painters exposed to a mixture of organic solvents). The spiked blank urine samples with HA and MA were prepared freshly from the stock solutions before analysis. The chemical structures and other physicochemical properties of toluene and ethylbenzene metabolites are illustrated in Table 1.

### 2.3. Chromatographic Conditions

The HPLC system (Knauer, Germany) equipped with a K-1001 piston pump, A K-2600 spectrometer was used for analysis. The apparatus has D-14163 degasser, a 6-port/3-channel injection valve fitted with a 20 µL sample loop and a Chromgate Software (EZ-chrom Elite, Darmstadt, Germany). Separation was achieved on a Eurospher C18 column (25 cm × 4.6 mm i.d., 5 µm) with the mixture of water/methanol/acetic acid, 69:30:1 (*v*/*v*/*v*) as the mobile phase. The elution was carried out under isocratic mode at a flow rate of 1.0 mL·min^−1^ and the UV detection wavelength was specified at 247 nm in a 20 μL injection volume.

### 2.4. Extraction Procedure by HF-LPME

The hollow-fibers were manually cut in to 8.8 cm equal pieces carefully, in which their internal volumes were 24 µL. Then, these pieces were washed with acetone in an ultrasonic bath for 5 min to remove any contaminants and dried in air. A 25 µL Hamilton HPLC-syringe was utilized to support the hollow-fiber and injection syringe. Also, it was applied to introduce the acceptor phase into the lumen of the hollow-fibers. Before the extraction process, 24 µL of acceptor phase (different solvents) was withdrawn into a 25 µL syringe and the end of the hollow fiber was immersed in organic solvent for 15 s to fill its pores with organic solvents. Next, in order to remove excessive organic solvents from their surfaces, the hollow-fibers were rinsed with distilled water for 10 s. Then, the acceptor phase in the microsyringe was carefully injected into the hollow-fiber and one end of it was closed with a segment of aluminum foil. For each experiment, a sample vial (12 mL) with a 14 mm × 4 mm magnetic stirring bar containing 10 mL of the aqueous sample (spiked and real sample urine) was placed on a VELP Scientifica heating magnetic stirrer (Milano, Italy), model ARE (Milan, Italy) for sample agitation. Then, the hollow-fiber was bent into a u-shape configuration and introduced into the donor solution. After the extraction, within the given period of time, the end of the HF was cut and the acceptor solution containing the analytes of interest was withdrawn into the syringe and injected into the HPLC system for analysis. Due to the low cost of hollow-fibers for elimination of memory effect, each piece was used only for one extraction. The concentration of the target metabolites in optimization experiments were 1 mg·L^−1^. Each extraction was performed for at least three replicate experiments.

## 3. Result and Discussion

### 3.1. Effect of Extraction Solvent

**Figure 1:**
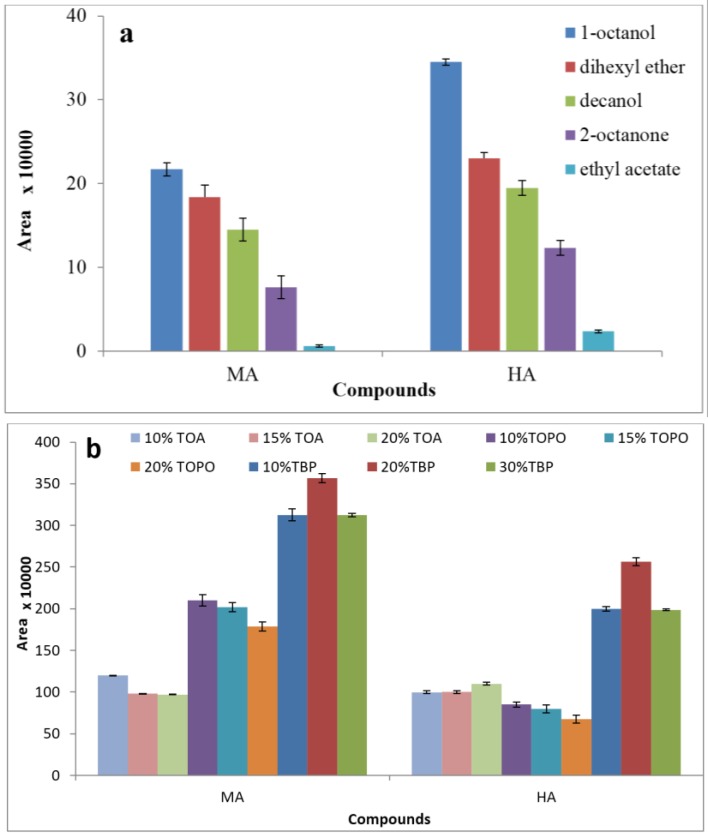
Effects of organic liquid membrane (**a**) and type and amount of carrier (**b**) on the extraction efficiency of hippuric acid (HA) and mandelic acid (MA) by hollow-fiber liquid-phase microextraction (HF-LPME). Extraction condition: 10 mL aqueous sample solution of 1 mg·L^−1^ metabolites (pH = 4); 24 µL acceptor phase sodium carbonate (pH = 10); stirring rate: 800 rpm; donor temperature 25 °C; 30 min extraction time and each test was performed in triplicate (*n* = 3). TOA: trioctylamine, TBP: tributyl phosphate, TOPO: trioctylphosphine oxide.

Five different organic solvents functioning as the supported liquid membrane, including decanol, 1-octanol, 2-octanone, ethyl acetate, and dihexyl ether, were investigated for extraction efficiencies. The best extraction performance was obtained using 1-octanol in terms of analyte peak area (Figure 1a). An effective organic solvent functioning as the supported liquid membrane should be suitable for hollow-fiber, so that all the pores in the wall of the fiber can be loaded and easily completely immobilized. Therefore, it must be water-immiscible when employed as a barrier between two donor and acceptor phases. It is important that an organic solvent must have a good tendency to dissolve target analytes and low viscosity to increase mass transfer through the membrane and low volatility to prevent loss of solvent throughout the extraction process [34,35,36]. Under optimal conditions, in addition to pH gradients, carrier-mediated transport was used to promote the extraction efficiency.

Three different extractants with various concentrations as carriers, including TBP, TOPO, and TOA, were added to the 1-octanol held in the wall pores of the polymer hollow fiber to explore the effect of carriers on extraction efficiency. However, alkyl and alkoxy-phosphoryl carriers (TOPO and TBP) are known as efficient extractants for polar analytes like carboxylic acids because of their ability to form hydrogen bond complexes [37]. According to the results, 20% (*w*/*v*) TBP in octanol showed the best performance (Figure 1b) and it was selected as the extraction solvent in all subsequent experiments. 

### 3.2. Effects of Donor and Acceptor pH

**Figure 2:**
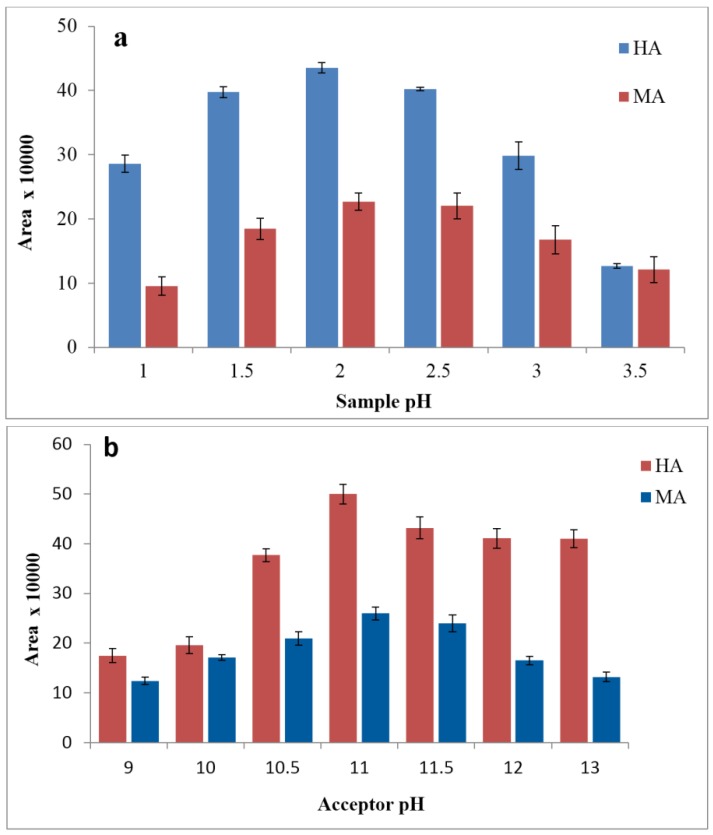
Effects of sample pH (**a**) and acceptor pH (**b**) on extraction efficiency of HA and MA by HF-LPME. Extraction conditions: 10 mL aqueous sample solution of 1 mg·L^−1^ metabolites organic membrane phase: 1-octanol; donor temperature 25 °C; stirring rate 800 rpm: 30 min extraction time and each test was performed in triplicate (*n* = 3).

To improve the target analyte recoveries, the pH of the aqueous donor and acceptor should be regulated to a value that ensures that the analyte exists in the proper form that will allow for the most efficient extraction by the organic phase [22,38,39]. In the three-phase HF-LPME technique, to run the mass transfer through the donor and acceptor phases, the analytes should be able to exist in non-ionized form on the donor phase, so that they migrate through the organic membrane and are in an ionic form on the acceptor phase to be fully trapped and thereby concentrated. In accordance with the rule of thumb, for the extraction of acidic analytes, the acceptor pH should be more than pk_a_ + 3.3 and the donor pH less than pk_a_ − 2 [40]. The hippuric acid and mandelic acid are both acidic compounds with a pk_a_ value of 3.68–3.75. They are charged in basic and neutral solutions and uncharged in an acidic solution. The effect of donor pH on the extraction efficiency was investigated in the range of 1–3.5. The expected pH value was adjusted by adding concentrated hydrochloric acid dropwise into the donor phase. The results shown in Figure 2a, indicated that the peak areas of the analytes virtually increased with increasing in donor pH from a value of 1 to 2. At pH higher than 2 the peak area for two metabolites were significantly decreased. Therefore, the pH 2 value was considered for the subsequent analysis. We also investigated sodium carbonate at concentrations in the range of 0.1–2 mol·L^−1^ to specify the appropriate acceptor phase. The best extraction performance was achieved by using a sodium carbonate concentration of 1 mol·L^−1^ (pH 11). Hence, it was selected for the acceptor phase in the studies (Figure 2b).

### 3.3. Effects of Agitation and Salinity

**Figure 3:**
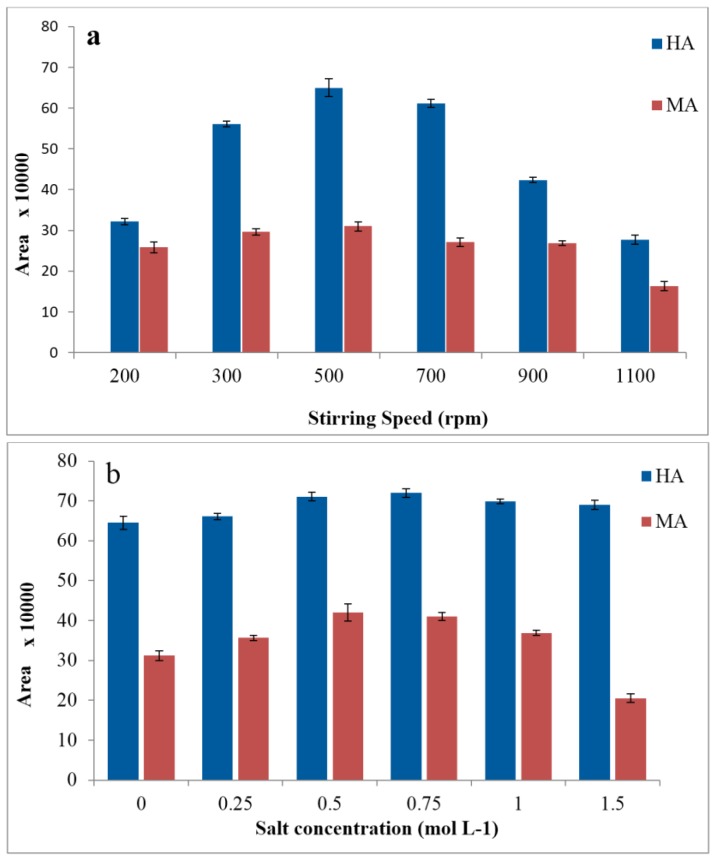
Effects of stirring speed (**a**) and salinity (**b**) on extraction efficiency of HA and MA by HF-LPME. Extraction conditions: 10 mL aqueous sample solution of 1 mg·L^−1^ metabolites with pH = 2; organic membrane phase: 1-octanol; 24 µL acceptor phase sodium carbonate (pH = 11); donor temperature 25 °C and each test was performed in triplicate (*n* = 3).

The sample stirring rate in hollow-fiber liquid-phase microextraction, as in other microextraction methods, can promote extraction efficiency so that it increases mass transfer and diffusion of the analyte from the donor phase into the acceptor phase and reduces the time required to achieve thermodynamic equilibrium. The production of excessive air bubbles on surface of fiber at high stirring speeds is not beneficial to the recoveries and promotes solvent evaporation [41]. In the present work, the stirring speed was studied from 200 to 1100 rpm for a 30 min extraction. Figure 3a shows that the efficiency increased with the stirring speed up to 500 rpm, while the extraction decreased at higher rates because at high stirring rates, air-bubbles are generated, which results in loss of solvent, thereby reducing the precision. Therefore, 500 rpm was selected as the optimized agitation speed for the rest of the experiments.

Commonly, salt addition can reduce the solubility of compounds in the aqueous sample and increase their transference into the organic phase. Although these effects have been discussed and there are some contradictory results, some experiments have mentioned that the addition of salt has a negative effect on extraction recovery as interaction between the analyte and salt leads to a decrease in diffusion rate of the analyte from the donor phase into the membrane organic solvent [29,30,42]. The effect of salt addition on the extraction efficiency of HA and MA were evaluated using solution containing 0–1.5 mol·L^−1^ of sodium chloride. The results showed that the efficiency enhanced with the increase of NaCl concentration up to 0.5 mol·L^−1^ and then decreased with further addition of the salt (Figure 3b).

### 3.4. Effects of Sample Temperature and Extraction Time

**Figure 4:**
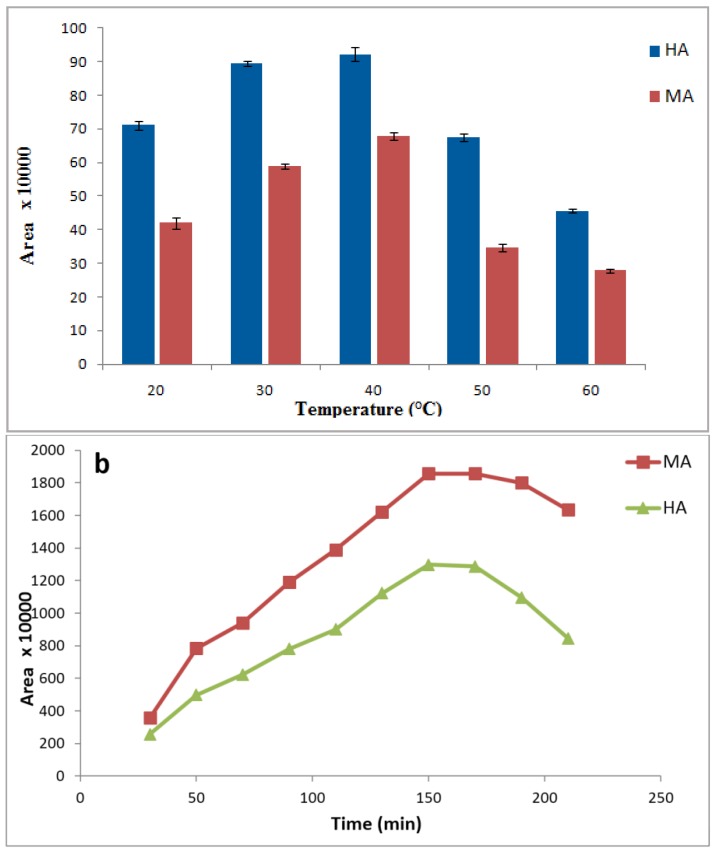
Effects of extraction temperature (**a**) extraction time (**b**) on extraction efficiency of HA and MA by HF-LPME. Extraction conditions: 10 mL aqueous sample solution of 1 mg·L^−1^ metabolites with pH = 2 containing 2 mol·L^−1^ NaCl; organic membrane phase: 1-octanol; 24 µL acceptor phase sodium carbonate (pH = 11); stirring rate: 500 rpm and each test was performed in triplicate (*n* = 3).

Temperature affects both kinetics and thermodynamics of the distribution process of the compounds during the extraction through the liquid phase microextraction method. Therefore, it can highly influence the extraction performance [43]. Meanwhile, loss of the organic phase occurs in the pores of hollow fiber at high temperature. In this study, the extraction efficiency increased with increasing the temperature up to 40 °C. Thus, this temperature was considered to be the best point for rest of the experiments (Figure 4a).

HF-LPME is an equilibrium process (non-exhaustive) and mass transfer is a time-dependent process in which analytes are partitioned between three phases, the aqueous donor matrix, the organic solvent and the aqueous acceptor phase, until equilibrium is established [44].

At equilibrium time, analytes in the receiving phase are in the steady state and stand at their maximum concentration. Moreover, extraction at a substantially longer time than equilibrium time can lead to a decrease in extraction recovery that may be due to loss of organic solvent in pores of hollow-fiber or receiving phase [17,18,41]. Thus, extraction time is one of the most important parameters affecting the extraction efficiency. As shown in Figure 4b, peak areas of target analytes increased with time extraction up to 150 min. Therefore, a sample extraction time of 150 min was selected for subsequent studies.

### 3.5. Method Validation and Application

Under optimized experimental conditions, 20% (*w*/*v*) TBP in 1-octanol as the organic membrane, sodium carbonate 1 mol·L^−1^ (pH 11) as acceptor phase and donor pH 2.0, stirring rate of 500 rpm, 0.5 mol·L^−1^ NaCl in donor phase as salt addition, 150 min as the extraction time, and donor temperature 40 °C) the performance and practical applicability of the three-phase HF-LPME configuration for extraction of HA and MA were investigated. Method validation was separately evaluated on the spiked blank urine samples containing the desired amount of both metabolites at various concentrations as the donor solution. Calibration curves were created from analyzes of six concentration levels in spiked blank urine for two analytes. To evaluate figures of merit of the method, linearity, limit of detection (LOD), limit of quantification (LOQ), dynamic linear range (DLR), enrichment factor (EF), repeatability, reproducibility and relative recovery were calculated. As can be seen in Table 2, the spiked blank urine calibration curves for HA and MA were detected to be linear in the range 0.02–20 and 0.02–10 mg·L^−1^ with determination coefficients (*R*^2^) of 0.998 and 0.997 for hippuric and mandelic acids respectively. The LODs—considered as the lowest concentrations of metabolites that give chromatographic signal-to-noise ratio of 3:1—were acquired in the range of 0.007–0.009 mg·L^−1^. The enrichment factor was calculated by the following equation:

EF = *C*_a, final_/*C*_a, initial_ (1)

where *C*_a, final_ and *C*_a, initial_ are the final metabolites concentration in the acceptor phase and their initial concentration in the donor sample, respectively. Under optimal conditions, the EFs were obtained at 172–195. The within-day precision (intra-day precision) was investigated at low, medium, and high concentration levels and with six replications for each. The day-to-day precision (inter-day precision) was performed on three consecutive days at the three concentrations of the target metabolites with eighteen replications for each in relative standard deviation. As shown in Table 2, the relative standard deviation (RSD)% of intra-day and inter-days were calculated 2.9%–8.2% and 4.1%–10.7%, respectively. Also, relative recovery (RR) is specified as the ratio of the concentration of the metabolite in the donor solution, determined with the proposed method, to the initial concentration of the metabolite in the sample solution, which was obtained at 88%–91% for HA and 84%–94% for MA.

The results of validation experiments indicated that the HF-LPME–HPLC method is suitable and feasible for the analysis of HA and MA in urine. 

### 3.6. Analysis of Real Samples Applicability

In order to apply the proposed extraction method for biological monitoring of toluene and ethylbenzene exposure, nine urine samples were obtained from volunteers at three different work environments in the cities of Hamadan and Assaluyeh, Iran: gas station, car painting and petrochemical plant, and the analytes in these real samples were determined. Under the optimized conditions, after appropriate dilution with deionized water, the metabolites were extracted and analyzed using the suggested method. The results are presented as urinary concentration ratio of metabolites to creatinine (mg/g creatinine). Since concentration of these analytes in urine is influenced by urine dilution, it was modified for urinary dilution using urinary creatinine concentration. Urinary creatinine was calculated in each urine sample using HPLC-UV. The results of three replicate analyses of each real urine sample have been reported in Table 3.

**Figure 5:**
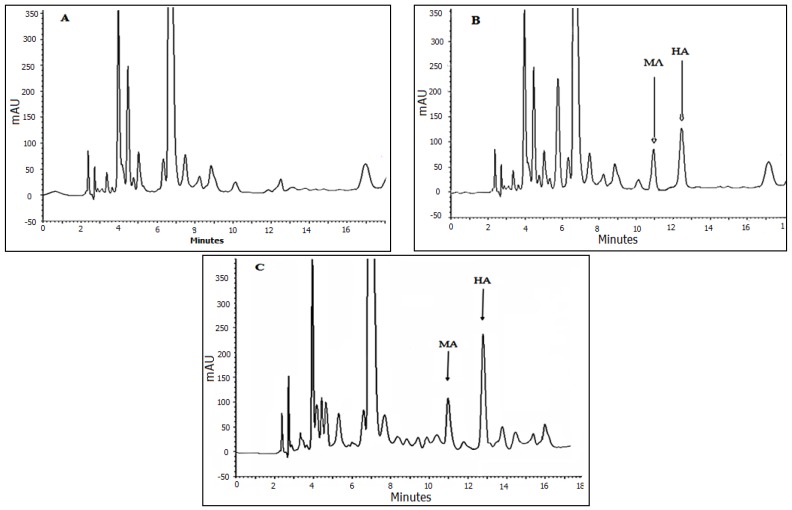
HPLC chromatograms of HA and MA after extraction under the optimized conditions as described in the text. (**A**) Blank urine sample from a person not exposed to benzene source; (**B**) spiked urine sample of the metabolites, at the concentration of 0.3 for ttMA and 1.5 µg·mL^−1^ for MA and HA; and (**C**) real sample from a person exposed to organic solvent source (petrol station worker).

Also, Figure 5 shows typical chromatograms established by HF-LPME based on carrier mediated transport for the blank, spiked and real urine samples.

### 3.7. Comparison of the Proposed Method with Other Conventional Reported Methods

A comparison between the LODs, LOQs, and RSDs of the proposed method with other published methods in terms of validation and precision demonstrates that this method, along with its simplicity, has high sensitivity that is comparable to or better than that of other methods, and has an acceptable precision (Table 4). In comparison with traditional liquid–liquid extraction and solid-phase extraction, hollow fiber-liquid phase microextraction based on carrier-mediated transport is more sensitive (lower LOD) and RSD of the method is acceptable. Compared with derivatization, HF-LPME is simpler and more convenient with less solvent and sample consumption. Another advantage of LPME over other techniques for sample preparation of urinary hippuric and mandelic acids is that the small pore size ensures microfiltration, thus yielding very clean extracts. For each extraction, a new piece of fiber was used so the possibility of carry-over was eliminated. Furthermore, this extraction technique provides efficient clean-up using only a small amount of organic solvent, which expected to be an alternative method in the related fields.

## 4. Conclusions

In the present study, we proposed for the first time the use of a HF-LPME based on carrier mediated transport as an applicable sample preparation method for the quantitative determination of HA and MA in urine, which can be used as a useful technique to evaluate occupational and environmental monitoring of exposure to very low concentrations of toluene and ethylbenzene. In comparison with conventional extraction methods like LLE, SPE and derivatization for sample pretreatment of urine containing HA and MA as a metabolites of toluene and ethylbenzene, hollow-fiber liquid-phase microextraction coupled with HPLC-UV, as an interesting alternative, provides reliable precision and accuracy and is simple to use, as well as being a less expensive procedure to overcome difficulties. Also, this method presents a very high enrichment factor and efficient clean-up, with a very low limit of detection with wide dynamic linear ranges, implying great advantages over other sample preparation procedures. Due to the simplicity of the extraction procedure, to overcome the long time of extraction, multiple extractions can be accomplished in parallel by using a multi-stirrer. 

## Figures and Tables

**Table 1:** The structures and physicochemical properties of toluene and ethylbenzene metabolites.

Metabolite	Chemical Structure	Log p (O/W)	pk_a_	Solubility in Water (g·L^−1^)
Hippuric acid	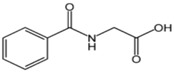	0.23	3.68	3.75
Mandelic acid	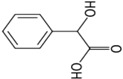	0.66	3.75	16.8

**Table 2:** Analytical performance of the proposed HF-LPME–HPLC method for extraction of hippuric acid and mandelic acid in urine.

Metabolite	Concentration (µg·mL^−1^)	Intra-Day RSD% *N* = 6	Inter-Day RSD% *N* = 18	LOD (µg·mL^−1^)	DLR (µg·mL^−1^)	EF	RR%	*R*^2^
HA	0.02	8.2	10.7	0.009	0.02–20	172	88–91	0.998
	0.2	5.6	7.2					
	2	2.5	4.1					
MA	0.02	2.9	7.5	0.007	0.02–10	195	84–94	0.997
	0.2	4.7	8.2					
	2	3.6	8.6					

**Table 3:** Concentrations of toluene and ethylbenzene metabolites detected in human urine of exposed workers.

No. of Sample Urine	Workplace	Mean ± SD (mg/g Creatinine)	
		HA	MA
1	Petrol station	‌376 ± 30	101 ± 10
2	Petrol station	483 ± 33	85 ± 9
3	Petrol station	221 ± 11	128 ± 12
4	Car painting	791 ± 78	180 ± 38
5	Car painting	344 ± 27	98 ± 38
6	Car painting	598 ± 47	125 ± 38
7	Petrochemical plant	287 ± 24	75 ± 21
8	Petrochemical plant	532 ± 42	121 ± 14
9	Petrochemical plant	435 ± 46	98 ± 18

**Table 4:** Comparison of the HF-LPME–HPLC method compared to other reported conventional methods for extraction and determination of MA and HA in human urine.

Analyte	Extraction	Determination	LOD (µg·mL^−1^)	LOQ (µg·mL^−1^)	RSD%	Ref.
HA	LPME	HPLC-UV	0.009	0.02	2.5–10.7	Proposed method
	Derivatization	GC-MS	0.017	0.05	6.2	[7]
	Dilution & filtration	HPLC-UV	1.5	4.5	0.2–0.5	[14]
	SPE	GC-MS	10	40	4.1–4.9	[2]
	SPE	LC/MS/MS	0.005	0.015	3–10	[13]
MA	LPME	HPLC-UV	0.007	0.02	3.6–8.6	Proposed method
	SPE	HPLC-UV	4	50	1.1–11.7	[15]
	Derivatization	GC-MS	0.008	0.05	7.7	[7]
	Dilution &filtration	HPLC-UV	7.6	22.8	0.4–2.6	[14]
	SPE	GC-MS	1	10	2.5–7.4	[2]
	LLE	HPLC-UV	5	–	3.9–4.4	[16]

GC-FID: Gas Chromatography-Flame Ionization Detector; GC-MS: Gas Chromatography-Mass Spectrometry; LC/MS/MS: Liquid Chromatography Mass Spectrometry and Liquid Chromatography Tandem Mass Spectrometry.
